# A Late Native Oesophago-Pulmonary Fistula, Oesophageal Foreign Body and Chronic Lung Abscess After Retrosternal Colo-Oesophagoplasty

**DOI:** 10.7759/cureus.64362

**Published:** 2024-07-11

**Authors:** Georgi Yankov, Magdalena Alexieva, Borislav Vladimirov, Mila Kovacheva-Slavova

**Affiliations:** 1 Thoracic Surgery Department, University Hospital St. Ivan Rilski, Medical University of Sofia, Sofia, BGR; 2 Gastroenterology, University Hospital Tsaritsa Ioanna-ISUL, Medical University of Sofia, Sofia, BGR

**Keywords:** fistula, surgical treatment, lung abscess, native oesophago-pulmonary fistula, retrosternal colo-oesophagoplasty

## Abstract

The development of a late fistula between a native unremoved corrosively altered oesophagus and the right lung with subsequent chronic lung abscess formation 34 years after retrosternal colo-oesophagoplasty is an extremely rare complication. According to our review of the English-language literature, such a case has not been described so far. We present a 50-year-old man with complaints of dry cough, periodic epigastric postprandial pain, regurgitation of food and halitosis, which started about seven years ago. Transthoracic right-sided subtotal oesophagectomy and resection of the sixth lung segment were performed. Diagnostics and surgical treatment are discussed.

## Introduction

The abnormal communication between oesophagus and the respiratory tract is divided into three types: tracheo-oesophageal, broncho-oesophageal, and oesophago-pulmonary fistula [1]. In addition, these fistulas are classified into congenital and acquired, malignant and non-malignant. The frequency of oesophago-pulmonary fistulas in both malignant and benign variants is about 3-11% [2]. Corrosive oesophago-bronchial fistulas have been also described [3]. A fistula between a native unremoved corrosive-altered oesophagus and the right lung with subsequent chronic lung abscess formation many years after retrosternal colo-oesophagoplasty is an extremely rare complication. Diagnostics is very difficult due to the impossibility of endoscopic examination of the left native oesophagus. Treatment is challenging and entirely surgical.

## Case presentation

**Figure 1:**
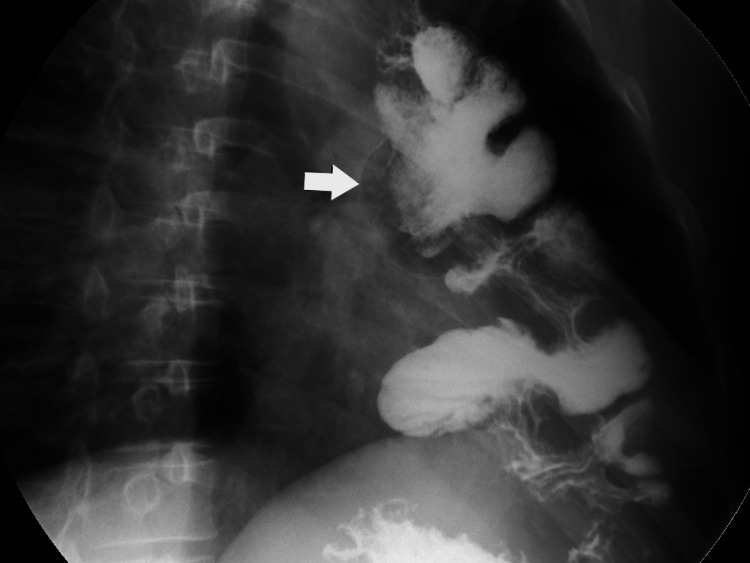
Contrast roentgenography, showing a well-functionating colo-oesohpagoplasty. The white arrow shows the intrathoracic retrosternal colon.

**Figure 2:**
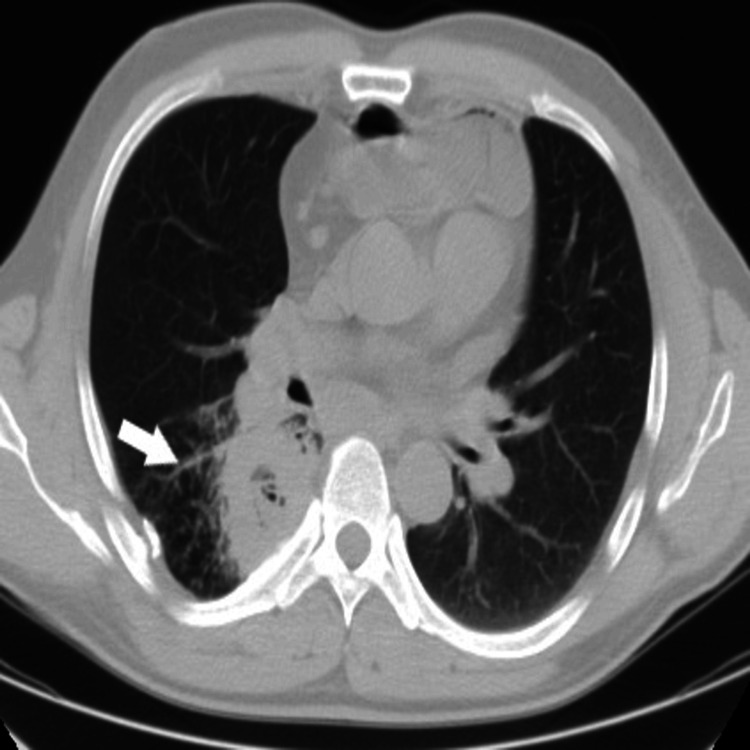
Computed tomography. Axial image of a right-sided lung abscess (white arrow).

**Figure 3:**
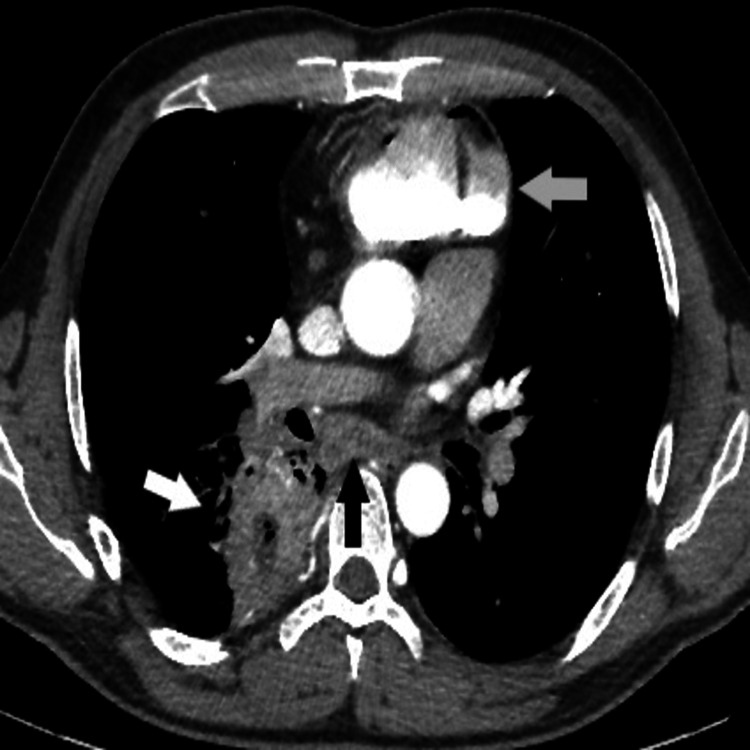
Computed tomography. White arrow- lung abscess, black arrow- oesophagus, grey arrow- contrasted retrosternal colon.

**Figure 4:**
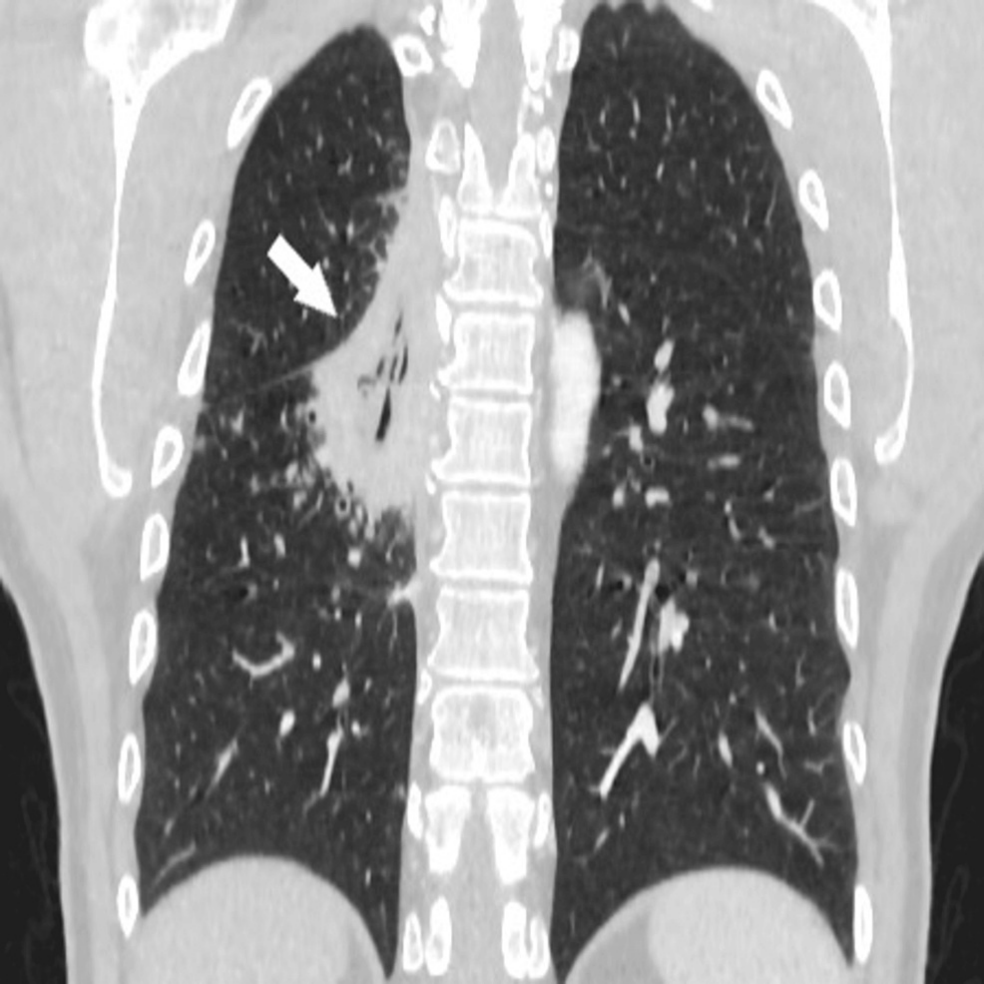
Computed tomography. Coronal images of lung abscess (white arrows).

**Figure 5:**
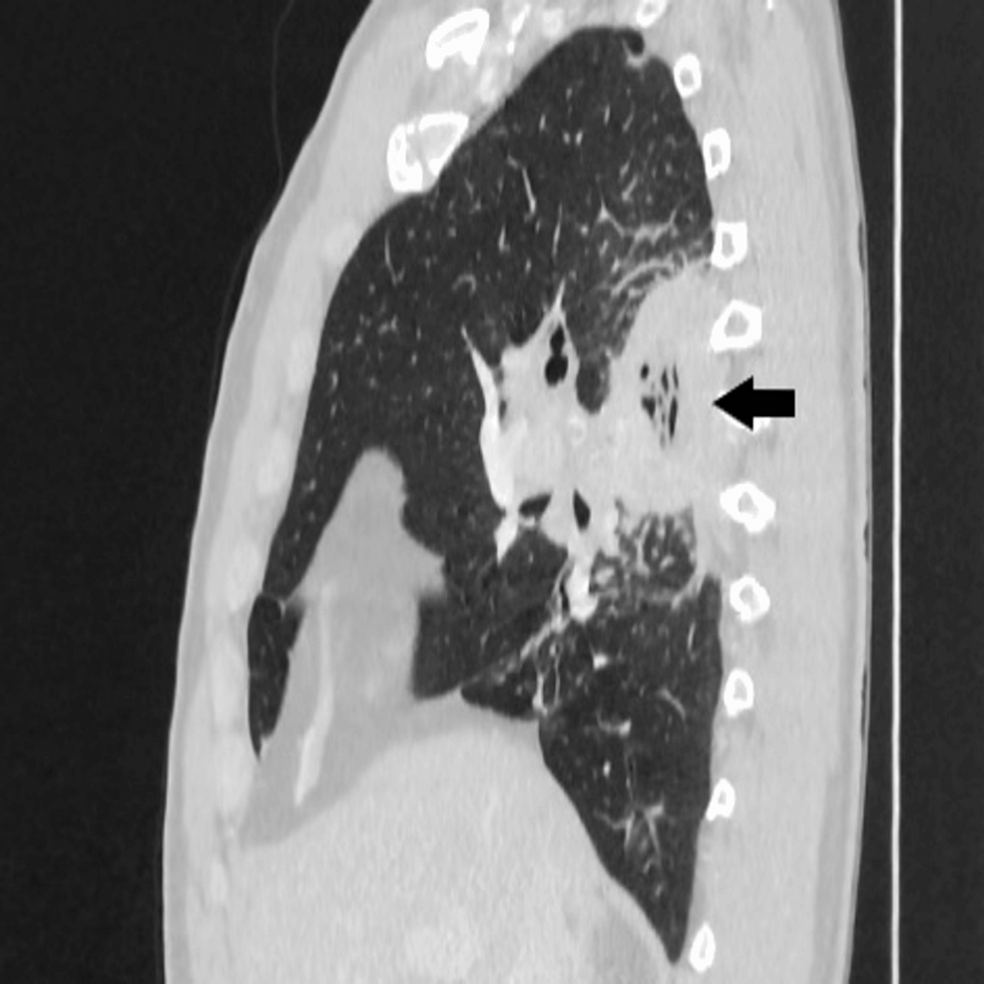
Computed tomography. Sagittal images of lung abscess (black arrow).

A 50-year-old man was admitted to Thoracic Surgery Department with complaints of dry cough, periodical epigastric heaviness after feeding, regurgitation of food and halitosis, which started approximately seven years ago. The patient had medical history of a corrosive oesophageal burn after accidental ingestion of caustic soda at the age of nine. Two years later, a post-corrosive stricture with 3 mm in diameter lumen in the area of second physiological esophageal stricture was observed. The patient underwent endoscopic dilatations with bougies and endoscopic balloon dilatation of the oesophagus. Furthermore, a Kader gastrostomy was performed. At the age of 13, after an iatrogenic oesophageal perforation, an emergency thoracotomy and oesophageal suture were carried out to the patient. All these procedures were executed in the Pediatric Surgery Department of another institution. Because of the persistence of post-corrosive stricture in the area of sutured section, three years later when the patient was 16 years old, in the Department of General Surgery of another institution, a bypass-type retrosternal colo-oesophagoplasty was performed. The native oesophagus however was not removed. One year later, two corrective interventions in the region of cervical oesophago-colo anastomosis followed due to stenosis. At the age of 20, laser ablation ensued in the area of recurrent stricture and a long-term effect was achieved in the patient. During the next year, a degastrostomy procedure was performed. The patient suffered from pneumonia at the age of 42, and recently after that, due to symptomatic epilepsy, magnetic resonance images (MRI) revealed a brain abscess. A left frontal craniotomy and evacuation of the abscess contents were urgently performed at department of neurosurgery. At the admission in our department the contrast study of oesophagus showed normally functioning oesophago-colic anastomosis at the level of T2-T3 and gastro-colic anastomosis (Figure 1). Chest computed tomography (CT) scan demonstrated the retrosternal colon into the anterior mediastinum, filled well with per oral contrast media. At the fifth minute of the scan, the stomach was not filled with contrast. Paravertebrally at the level of the sixth right lung segment, an irregularly shaped liquid collection containing air was visualized (Figures 2-5). It had thick walls and entered into the pulmonary parenchyma, adhering to the costal and interlobar pleura. Cranially, it was connected with the native oesophagus. Inflammatory changes by the "tree in bud" type and a limited zone of consolidation in the neighboring lung parenchyma were visualized, and the segmental bronchus was not observed free - probably because of retained secretion. CT evidence for a bony bridge between the right fifth and sixth ribs along posterior axillary line from the previous thoracotomy. An abscess was diagnosed, probably on the basis of a fistula between the right lung and oesophagus. Upper endoscopy was carried out and a pus-like collection on the anterior wall due to oesophago-pulmonary fistula was visualized about 25 to 30 cm from the dentition, which was partially aspirated. Fiberoptic bronchoscopy showed inflammatory process with a hyperemic, easily bleeding lower bronchus with uneven, granular and swollen mucosa, filled with purulent secretions. Histology resulted in papillomatosis of the lining epithelium, subepithelial pronounced chronic inflammatory infiltrate with capillary proliferation.

**Figure 6:**
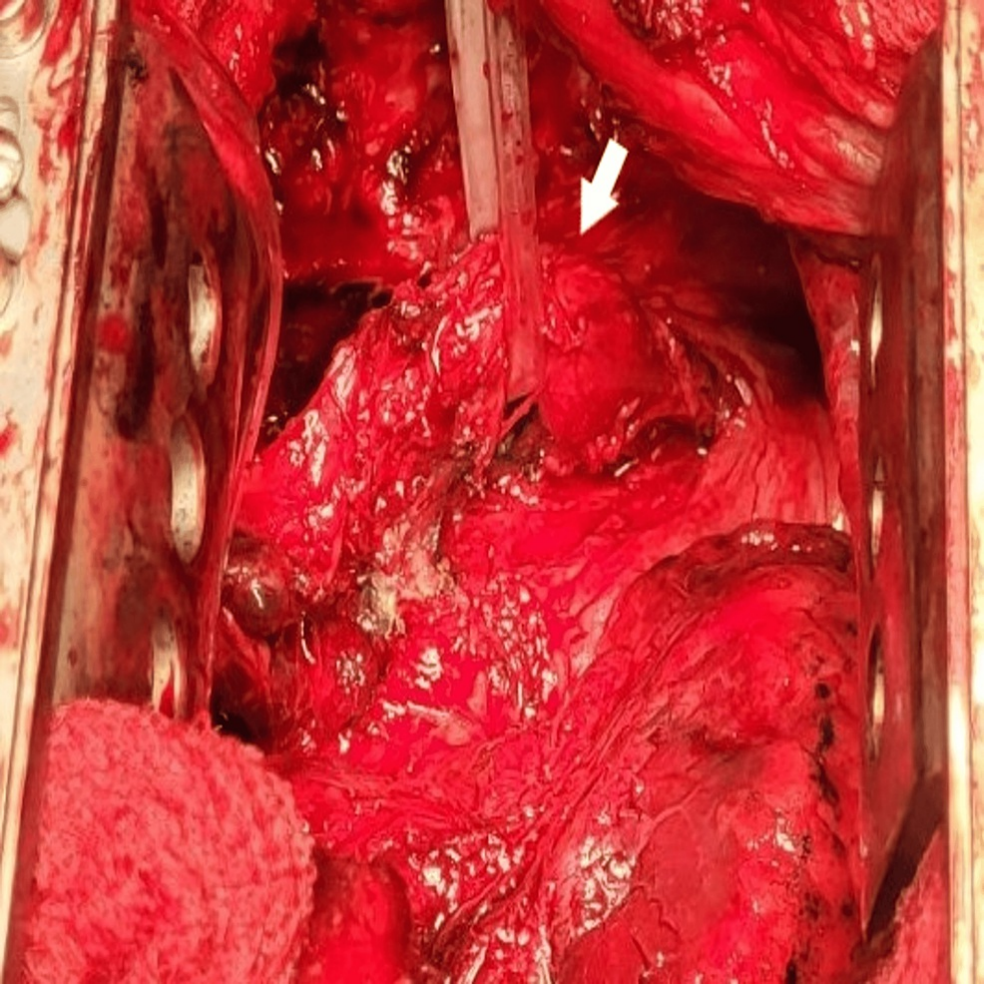
Intraoperative images. Oesophageal mobilization (white arrow).

**Figure 7:**
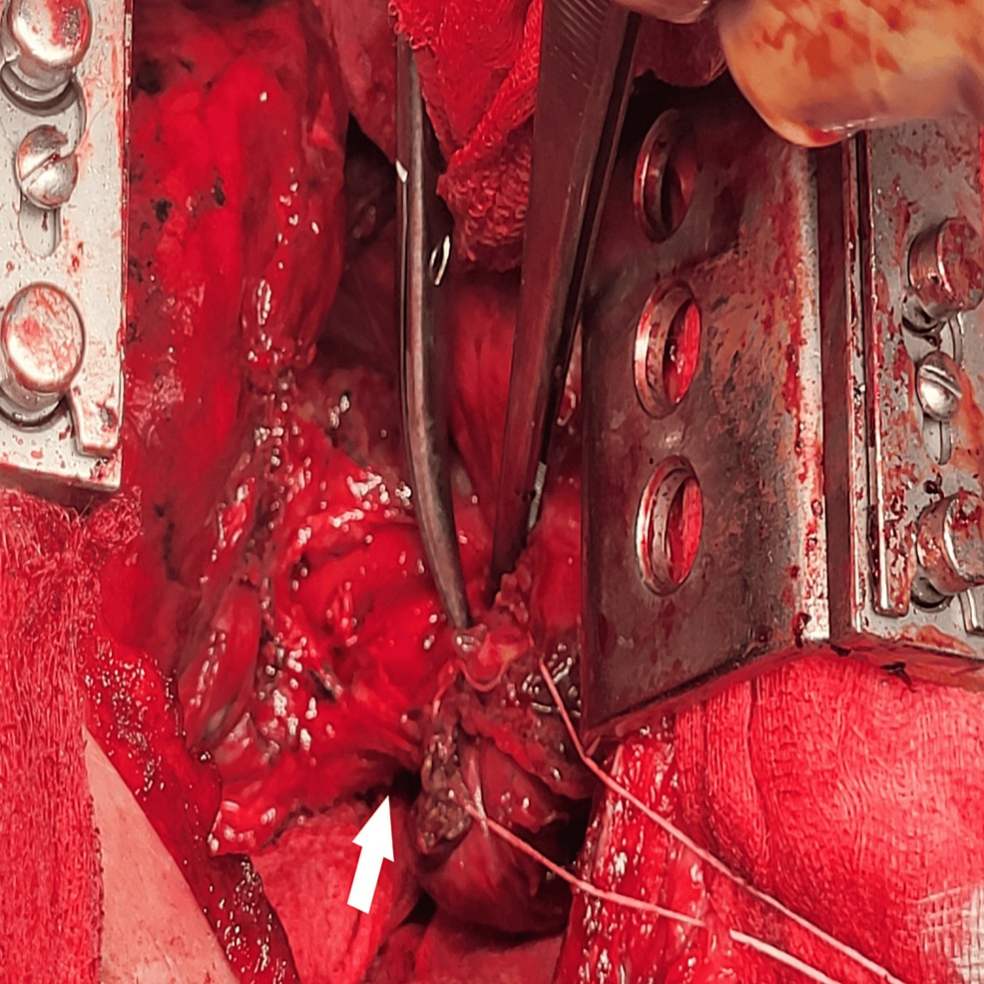
Intraoperative images. A fistula between the oesophagus and the sixth segment (white arrow).

**Figure 8:**
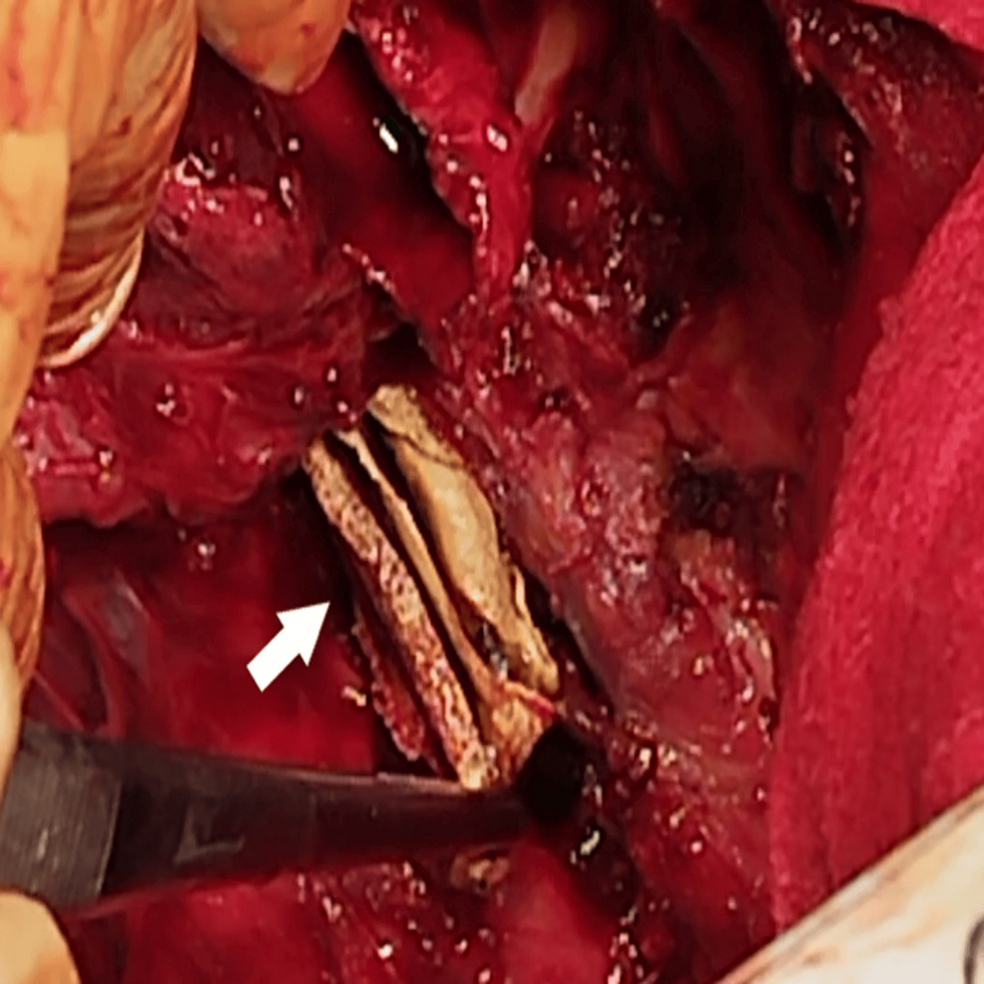
Intraoperative images. White arrows show taking out the oesophageal foreign body.

**Figure 9:**
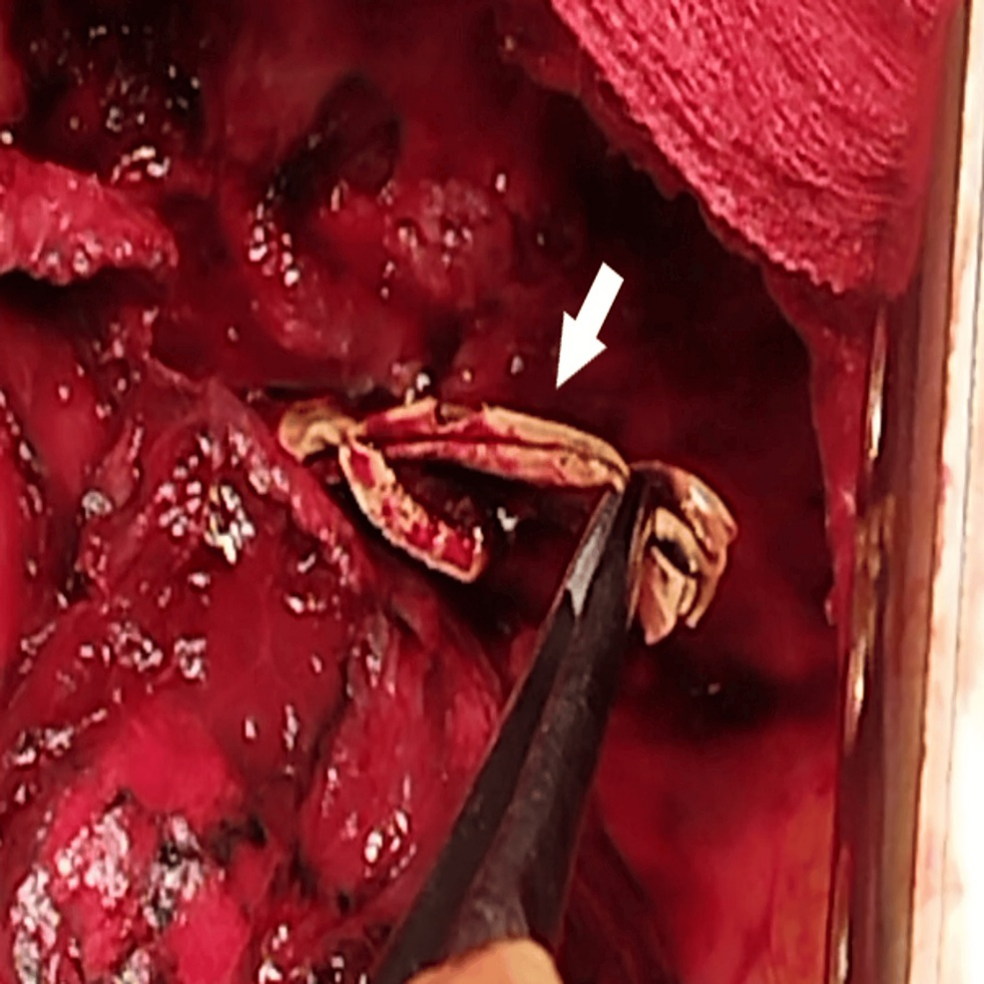
Intraoperative images. White arrows show taking out the oesophageal foreign body.

**Figure 10:**
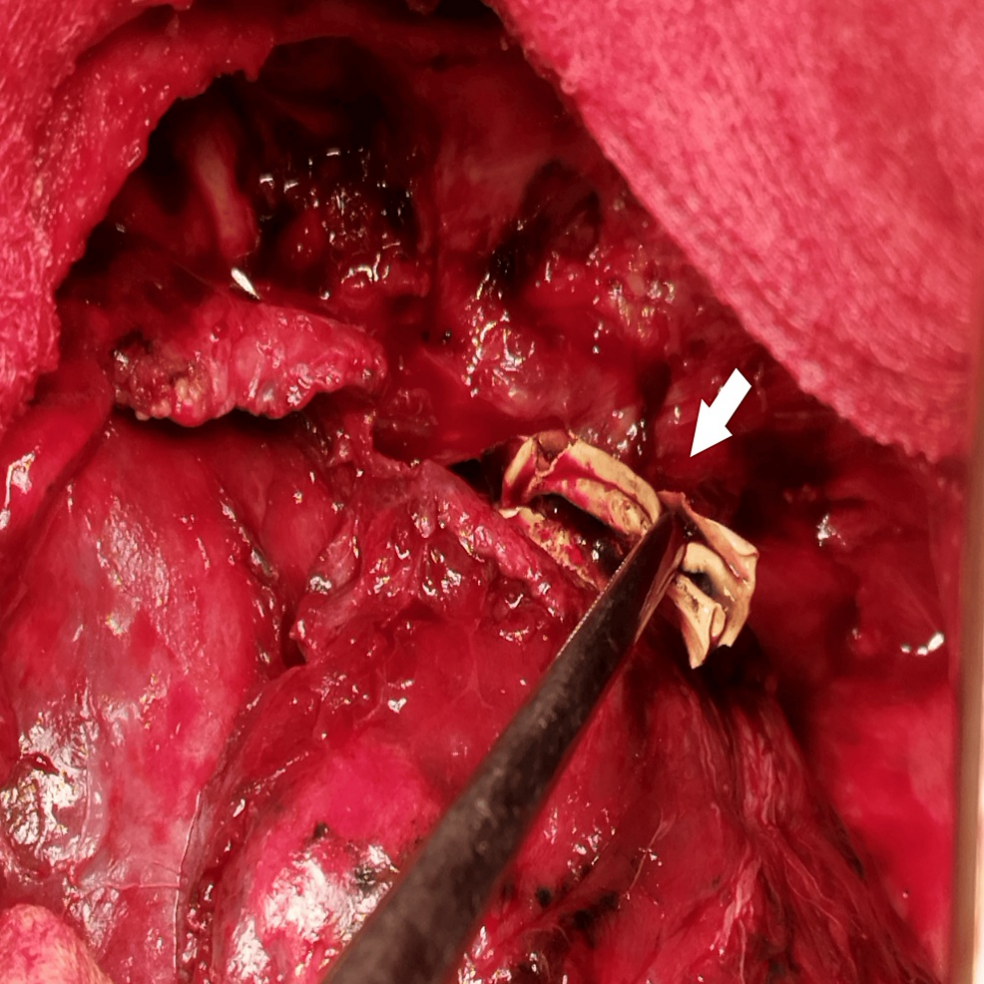
Intraoperative images. White arrows show taking out the oesophageal foreign body.

A right postero-lateral thoracotomy was performed and revealed partially obliterated pleural cavity due to the previous surgery. Multiple dense adhesions between the lung and the costal, mediastinal, and diaphragmatic pleura were debrided. We found a pathological communication between a chronic abscess in the sixth segment and the middle third of the native oesophagus, which was filled with pus (Figures 6-10).

**Figure 11:**
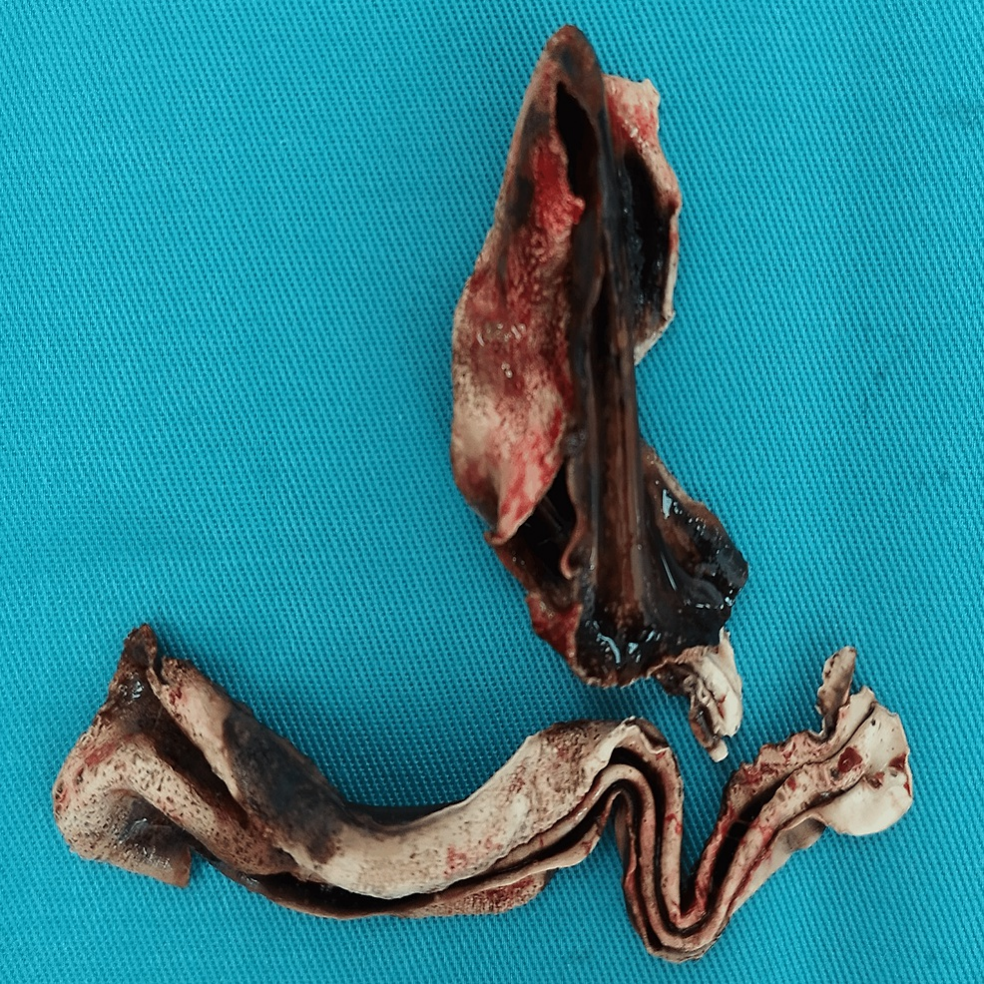
Postoperative photos of the foreign body in the native oesophagus that resembled altered and lysed Pezzer catheter.

**Figure 12:**
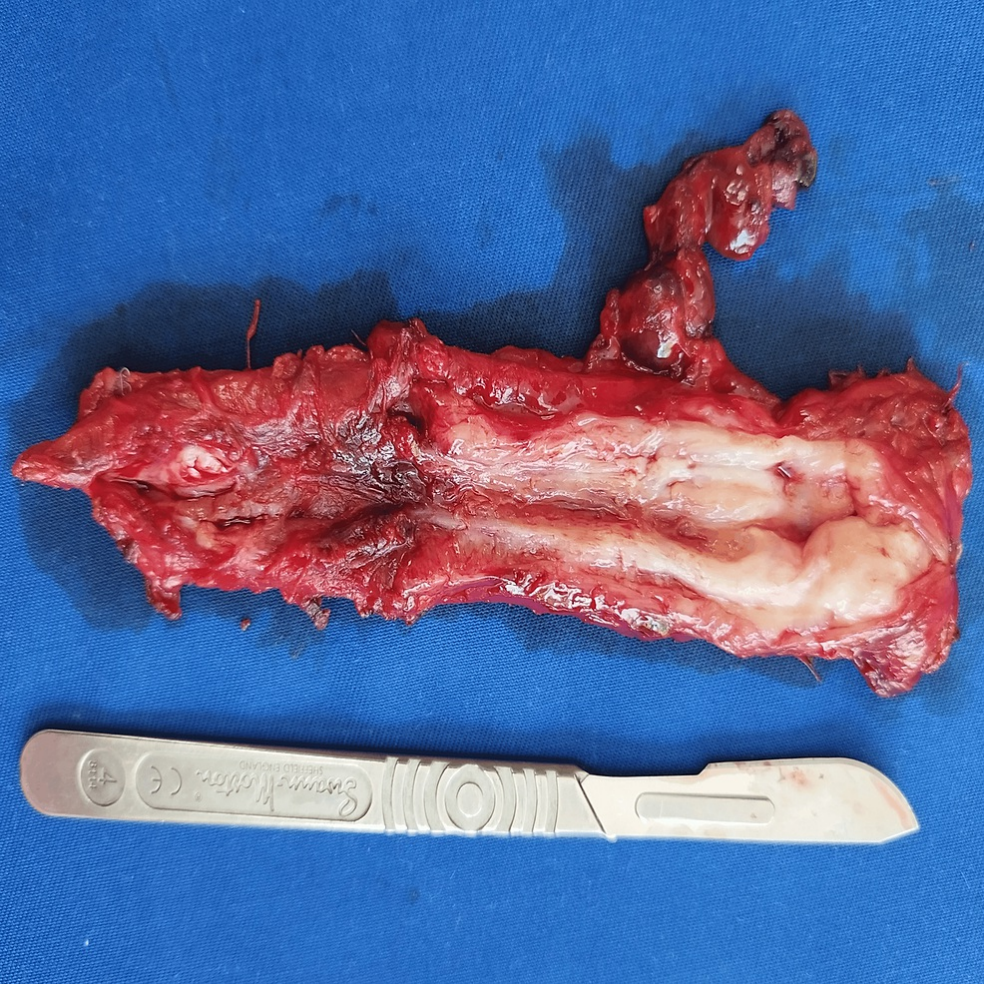
Postoperative photos of the specimens. A section of the removed oesophagus - evidence of a chronic corrosive stricture, and in some areas the lumen was missing.

**Figure 13:**
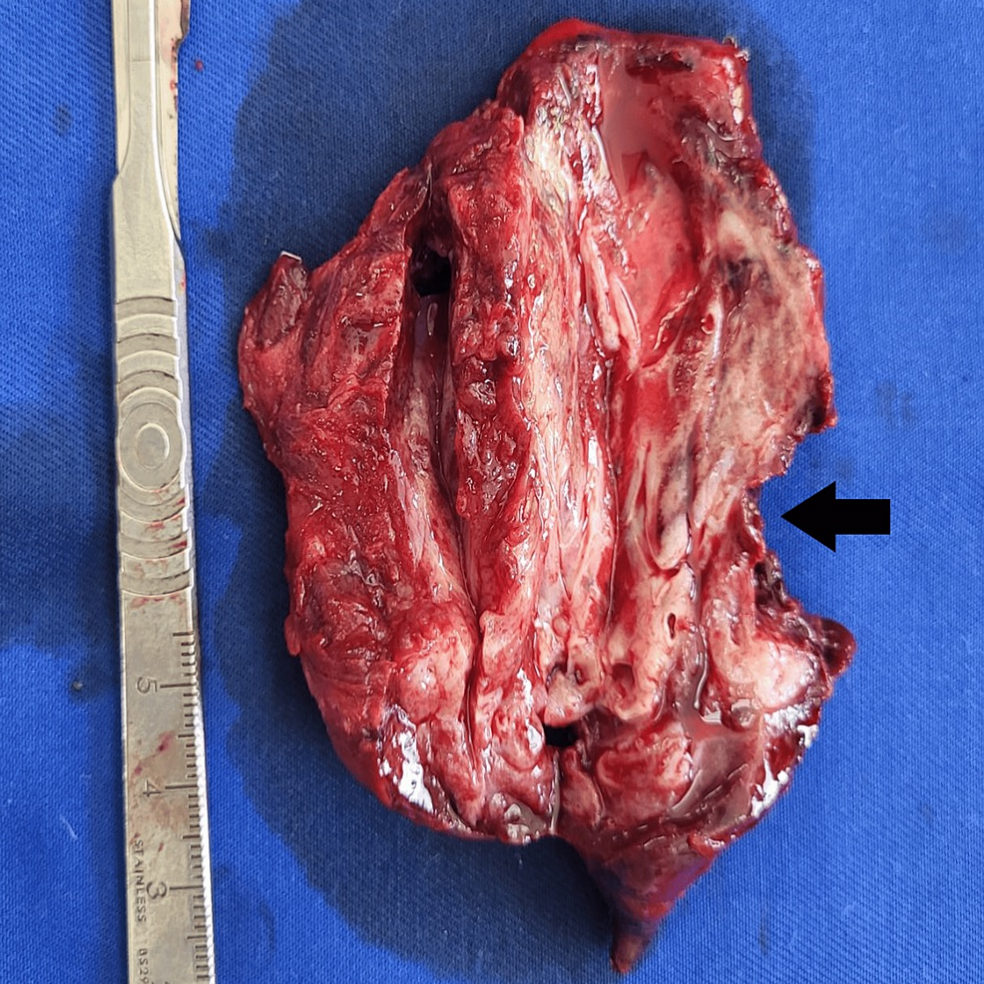
Postoperative photos of the specimens. A section of the resected sixth segment - an abscess cavity.

A fragmented bisected plastic band-like material resembling a highly altered and lysed endoluminal Pezzer catheter was removed from the esophageal lumen (Figure 11). The mediastinal pleura was incised from hiatus to the upper thoracic aperture. The walls of the oesophagus were greatly thickened with a massive periesophagitis (Figures 12, 13). The azygos vein was ligated and transected. We performed subtotal oesophagectomy, cutting towards cardia and at the level of the upper thoracic aperture with two staplers. An anatomical resection of the sixth segment was carried out. The hilar elements were processed in the following order: the artery and vein were ligated, the bronchus for the sixth segment was stapled, and the remaining lung parenchyma was also stapled. Several anthracotic inflammatory-changed lymph nodes located around the lower lobe vessels were also excised.

Pathology of the specimen showed oesophageal wall with epidermoid metaplasia of the lining mucosa ("oesophageal leukoplakia"), transition to Barrett's mucosa without dysplasia and areas of superficial ulceration, subepithelial mixed-cell inflammatory infiltrate with plasma cells dominance, proliferation of capillary vessels along the course of the entire wall, fibrosis with hyalinization of the wall and adventitia. In the area of the oesophago-pulmonary fistula - lung parenchyma with abscess, peribronchial and bronchiolar inflammatory infiltrates, mainly represented by lymphocytes and plasma cells, interstitial fibrosis and, against this background, reactive proliferation of pneumocytes with intraalveolar accumulation of macrophages. Presence of pulmonary hamartoma. All 11 dissected lymph nodes were with signs of chronic sinus lymphadenitis. 

The postoperative period was uneventful and the patient was discharged on the 10th postoperative day. Six months later the patient is in an excellent general condition and without any complaints.

## Discussion

It is well known that acids cause coagulation necrosis that prevents deeper tissue damage, however the basic substances create liquefaction necrosis that create full-thickness injury [4]. Surgical treatment of severe corrosive strictures most often consists of colonic replacement [5]. Retrosternal interposition with colon is the safest option [6]. The usage of stomach as a graft is often impossible due to the synchronous oesophageal and stomach burn, and on the other hand, the jejunum is usually too short for performing neck anastomosis. In extensive caustic injuries the best functional results are accomplished by isoperistaltic left colon grafts [7]. We favor the use of left colon that is translated both retrosternally or posteromediastinally over the course of the native oesophagus after its removal. In corrosive stricture cases we obligatory remove the native oesophagus. Oesophageal resection could be done at the first stage by taking out oesophagostomy and gastrostomy until the patient's condition is stabilized and the nutritional deficiency is corrected. Subsequent retrosternal replacement with a colon through a second-stage surgery follows. We can also perform oesophagectomy (very often transhiatally) and colon replacement in one act, using the route of the native oesophagus (posteromediastinal). The complications after colo-oesophagoplasty might be early (colon necrosis, arterial or venous impairment, anastomosis dehiscence) and late (fistula, stenosis, colon redundancy, peptic ulceration, reflux, cancer, dysfunctional disorders as dysphagia, etc.) [4].

We present a rare late oesophago-pulmonary fistula, which is extremely serious and is apparently due to the non-removing the native oesophagus in a young patient with expected long survival. Multiple bougineges, high-grade stricture, and subsequent suturing of the resulting iatrogenic perforation are likely key factors for the development of this complication. Chronic irritative changes to the mucosa of the native oesophagus also contributed to the formation of a fistula and chronic lung abscess. Other described uncommon complication of the excluded non-resected oesophagus is mucocele [4]. 

In our case, the surgery was extremely difficult and challenging because of the massive adhesions and the altered pleural and mediastinal anatomy from the previous operation. Severe peri- and panoesophagitis accompanying corrosive burns further hinder dissection of the oesophagus from the surrounding mediastinal structures. We removed a fragmented in two parts plastic band-like material from the oesophageal lumen, resembling a highly modified and lysed endoluminal Pezzer catheter. Probably the latter was used through the endless bouginages through the previous gastrostomy and was left there to serve like a stent. Such a technique was used in the late 1980s when today's modern dilators and stents did not exist. Removal of the native oesophagus was mandatory. Due to localized changes only in the sixth pulmonary segment, only segmental resection of the latter was performed. Eight years before our intervention at the age of 42, an emergency craniotomy with evacuation of a brain abscess was performed on our patient due to hematological spread right-sided pneumonia. Leaving a corrosively altered oesophagus in young patients with expected long survival is also associated with another late complication, such as malignancy of the corrosive stricture. The risk of developing oesophageal carcinoma is about 2 to 30% and occurs between 10 and 30 years after ingestion of a corrosive agent [8]. Its diagnosis is extremely difficult due to the impossibility of endoscopic examination. Patient follow-up is mandatory due to increased risk of cancer development [9]. Despite periodic CT scans, malignancy of the stricture at an earlier stage could be missed and the diagnosis may remain unclear.

## Conclusions

Late fistula between the native oesophagus and the right lung with subsequent chronic lung abscess is a rare complication occurring many years after retrosternal colo-oesophagoplasty. According to our review of the literature, such case has not been described so far. Treatment is challenging and mainly surgical. Oesophageal resection is mandatory either in one stage (oesophagectomy with colon replacement) or in two-stage surgery (oesopgahostomy, gastrostomy, followed by oesophago-colopasty).
